# Effect of an Intrapartum Pelvic Dilator Device on Levator Ani Muscle Avulsion During Primiparous Vaginal Delivery: A Pilot Randomized Controlled Trial

**DOI:** 10.1007/s00192-024-05881-6

**Published:** 2024-08-03

**Authors:** Helai Hesham, Francisco Orejuela, Kara M. Rood, Mark Turrentine, Brian Casey, Meena Khandelwal, Rori Dajao, Sarah Azad, Todd Rosen, Matthew K. Hoffman, Eileen Y. Wang, Laura Hart, Jean-Ju Sheen, Tamara Grisales, Kelly S. Gibson, Vanessa Torbenson, Shauna F. Williams, Edward Evantash, Hans P. Dietz, Ronald J. Wapner

**Affiliations:** 1 Department of Obstetrics & Gynecology, Columbia University Irving Medical Center, New York, NY USA; 2 Department of Obstetrics & Gynecology, Baylor College of Medicine, Houston, TX USA; 3 Department of Obstetrics & Gynecology, Wexner Medical Center, Ohio State University, Columbus, OH USA; 4 Department of Obstetrics & Gynecology, University Medical Center, University of Alabama, Tuscaloosa, AL USA; 5 Department of Obstetrics & Gynecology, Cooper University Hospital, Camden, NJ USA; 6 El Camino Women′s Medical Group, El Camino Health, Mountain View, CA USA; 7 Department of Obstetrics, Gynecology and Reproductive Medicine, Robert Wood Johnson Medical School, Rutgers University, New Brunswick, NJ USA; 8 Department of Obstetrics and Gynecology, Christiana Care Health System, Newark, DE USA; 9 Department of Obstetrics & Gynecology, Hospital of the University of Pennsylvania, Philadelphia, PA USA; 10 Department of Obstetrics, Gynecology and Reproductive Medicine, Temple University, Philadelphia, PA USA; 11 Department of Obstetrics & Gynecology, University of California-Los Angeles, Los Angeles, CA USA; 12 Division of Fetal Maternal Medicine, The Metro-Health System, Cleveland, OH USA; 13 Department of Obstetrics & Gynecology, Mayo Clinic, Rochester, MN USA; 14 Department of Obstetrics, Gynecology and Reproductive Health, Rutgers New Jersey Medical School, Newark, NJ USA; 15 Materna Medical, Mountain View, CA USA; 16 Sydney Urodynamic Centres, Sydney, NSW Australia

**Keywords:** Intrapartum pelvic dilation, Levator ani avulsion, Pelvic floor muscle injury, Primiparous vaginal delivery

## Abstract

**Introduction and Hypothesis:**

The objective was to evaluate the safety and effectiveness of an intrapartum electromechanical pelvic floor dilator designed to reduce the risk of levator ani muscle (LAM) avulsion during vaginal delivery.

**Methods:**

A multicenter, randomized controlled trial enrolled nulliparous participants planning vaginal delivery. During the first stage of labor, participants were randomized to receive the intravaginal device or standard-of-care labor management. The primary effectiveness endpoint was the presence of full LAM avulsion on transperineal pelvic-floor ultrasound at 3 months. Three urogynecologists performed blinded interpretation of ultrasound images. The primary safety endpoint was adverse events (AEs) through 3 months.

**Results:**

A total of 214 women were randomized to Device (*n* = 113) or Control (*n* = 101) arms. Of 113 Device assignees, 82 had a device placed, of whom 68 delivered vaginally. Of 101 Control participants, 85 delivered vaginally. At 3 months, 110 participants, 46 Device subjects who received full device treatment, and 64 Controls underwent ultrasound for the per-protocol analysis. No full LAM avulsions (0.0%) occurred in the Device group versus 7 out of 64 (10.9%) in the Control group (*p* = 0.040; two-tailed Fisher’s test). A single maternal serious AE (laceration) was device related; no neonate serious AEs were device related.

**Conclusions:**

The pelvic floor dilator device significantly reduced the incidence of complete LAM avulsion in nulliparous individuals undergoing first vaginal childbirth. The dilator demonstrated an acceptable safety profile and was well received by recipients. Use of the intrapartum electromechanical pelvic floor dilator in laboring nulliparous individuals may reduce the rate of LAM avulsion, an injury associated with serious sequelae including pelvic organ prolapse.

## Introduction

Vaginal delivery carries risk of injury to the pelvic floor anatomical structures [1] and this risk is highest in nulliparous individuals [2]. Damage to pelvic floor structural integrity increases the likelihood of developing serious sequelae including pelvic organ prolapse (POP) [3]. Specifically, injury to the levator ani muscle (LAM) complex during vaginal delivery occurs in 13‒36% of individuals after vaginal birth [4, 5] and is strongly associated with subsequent POP [6, 7]. Over half of women with LAM subsequently develop POP beyond the hymen within the first 6‒17 years from first vaginal delivery [3]. Crowning of the fetal head during delivery considerably stretches the LAM and can lead to detachment, or avulsion, of the puborectalis component from its origin at the inferior pubic ramus. Partial or complete LAM avulsion can be quantifiably diagnosed using tomographic ultrasound imaging [8, 9]. Currently, there are no decisive preventive approaches documented in the literature for reducing these injuries to the pelvic floor muscles during vaginal birth.

The current study investigated an intrapartum electromechanical pelvic floor dilator (IPD) that was specifically designed to address the biomechanical resistance of pelvic tissues during vaginal birth. Computer models and in vivo MRI estimate that the diameter of the fetal head is 2.5‒3.5 times the diameter of the urogenital hiatus through the LAM [10, 11]. This size disparity imposes a remarkable degree of circumferential stretch on the LAM during crowning of the fetal head during the second stage of labor. The combination of rapid and excessive stretching risks LAM injury by exceeding the muscle’s elastic limit. To counter this effect, the IPD slowly stretches the vagina and surrounding pelvic tissues during late first-stage labor, thereby pre-acclimating the LAM and other pelvic floor components to the strain of crowning. A prior study confirmed the feasibility of incorporating an intrapartum vaginal dilator into labor and delivery care [12]. This study tested the hypothesis that the IPD device is a safe and effective intervention to reduce LAM avulsion incidence during first vaginal delivery.

## Materials and Methods

### Study Design and Participants

This randomized, controlled pilot study evaluated the safety and effectiveness of, and patient satisfaction with, an IPD designed to reduce the incidence of LAM avulsion during vaginal birth. The study was performed at 15 tertiary medical centers in the USA. Enrollment spanned September 2021 to September 2022. Participants were randomized 1:1 during the first stage of labor to either receive the IPD (Device arm) or standard of care (Control arm), in accordance with a computer-generated randomization sequence (www.randomize.net). Institutional review board approval was obtained for all sites, as a nonsignificant risk study. Reporting adhered to CONSORT guidelines. Study sites were chosen whose enrollment would mirror the diverse background of the contemporary USA populace [13, 14]. All participants provided written informed consent; patient education materials and consent forms were available in Spanish and English.

### Inclusion/Exclusion Criteria

Primary inclusion criteria were nulliparous individuals aged ≥ 18 years planning first singleton vaginal delivery at term (> 36 weeks), with willingness to receive epidural anesthesia prior to randomization. Primary exclusion criteria were fetal chromosomal or structural anomalies, local or systemic infection, maternal history of connective tissue disorders or neurological disease that could impact delivery, or unresolved intrapartum category 2‒3 fetal heart tracing prior to randomization.

### IPD Device and Procedure

**Fig. 1:**
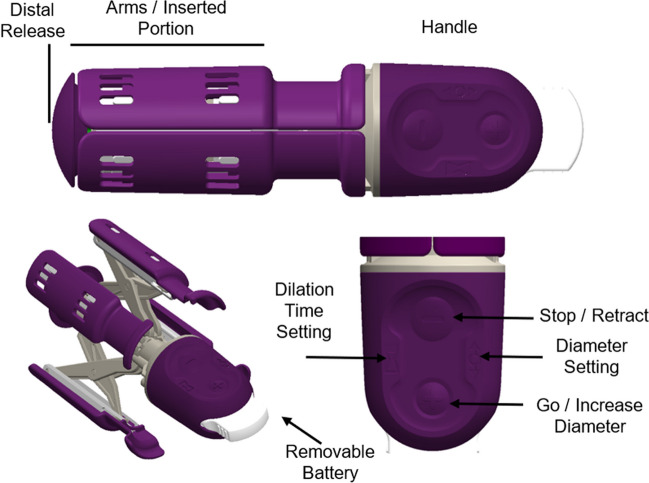
The intrapartum electromechanical pelvic floor dilator (*IPD*) device. Pre-deployment, the device is 12.6 cm long and 3.4 cm in diameter. The device is inserted 4‒5 cm into the vagina when the patient is in active labor, with at least 1 h expected before the second stage of labor. The four arms of the IPD expand outward in regulated increments to achieve gradual expansion from 3.4 cm to 8.0 cm over 60 min

The electromechanical IPD is a single-use intravaginal device (Fig. 1). The device gradually expands the vaginal introitus and pelvic tissues from a resting diameter of 3.4 cm to 8.0 cm over ≈60 min. The final preset diameter of 8.0 cm is 1‒2 cm smaller than the average fetal biparietal diameter [15]. Device placement occurred during active first stage of labor, with more than 1 h estimated remaining until complete cervical dilation. Device expansion was controlled by a mechanism that is designed to expand slowly at a constant rate, thereby gently prestretching the vagina and surrounding pelvic muscles. The device could be stopped and quickly retracted by the operator at any time and for any reason (e.g., to perform cervical examinations). Device diameter was displayed on the handle. The device was force limited and would not continue expansion if the vaginal wall/pelvic floor pressures exceeded a predefined safety threshold. A pressure-sensitive mechanism on the distal tip of the device is activated if there is tissue contact with the device, notifying the clinician that treatment may need to be discontinued if the descending fetal head is touching the device. After IPD therapy was complete and the device reached maximal dilation, the device arms were retracted and the device removed. If the second stage of labor had not ensued within 3 h of removal, treatment could be repeated at the discretion of the clinician.

All investigators were trained in IPD use and study protocols. Per protocol, all participants received epidural anesthesia prior to randomization. Reasons for not employing the IPD in participants randomized to the Device arm included, for example, rapid cervical dilation and changes in fetal heart tracing. After device removal, inspection of the vagina and perineum was performed to identify any lacerations or bleeding. Participants in both groups who underwent Cesarean section (C-section) after randomization were withdrawn from the study.

### Follow-up Schedule

Participants were asked to return 3 months after vaginal delivery for ultrasound examination, inquiry into symptoms and quality of life using the Pelvic Floor Impact Questionnaire-7 (range 0‒300 points; 300 = maximum negative impact) and the Pelvic Floor Distress Inventory-20 (range 0‒300 points; 300 = maximum distress) tools [16], and were queried on general device-related satisfaction on a 1‒10 analog scale (10 = highest).

**Fig. 2:**
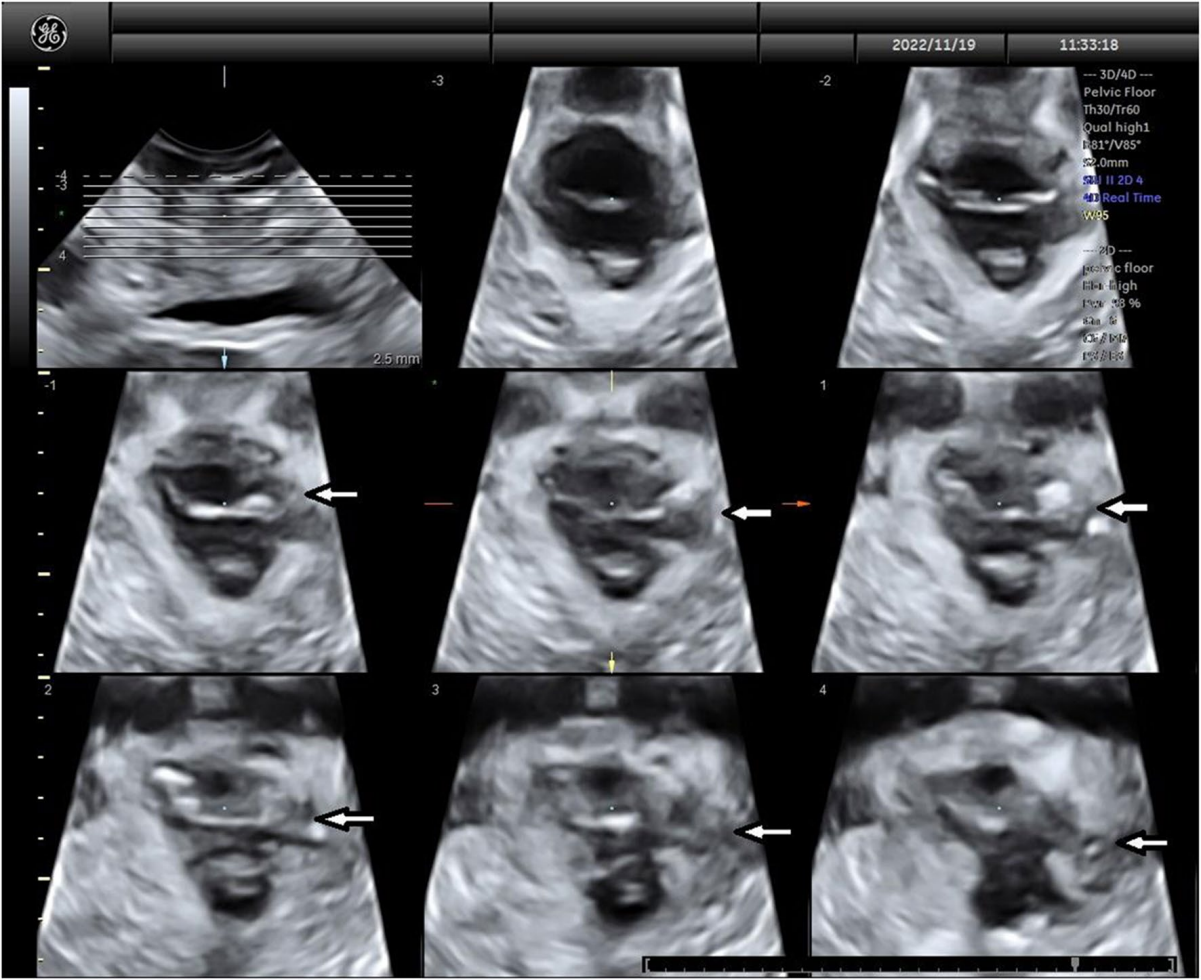
Transperineal tomographic ultrasound imaging of the levator ani muscle (*LAM*). Sequential-level ultrasound imaging reveals full left-sided avulsion (*white* *arrows*) of the levator ani pelvic floor muscle group 3 months after vaginal delivery. The avulsion injury appears as a frank interruption in the smooth arcing topography of the intact LAM complex seen on the contralateral (*right*) side in these ultrasound images

Ultrasound examination was performed in accordance with American Institute of Ultrasound in Medicine/International Urogynecological Association (AIUM/IUGA) practice parameters for urogynecological ultrasound examinations [17]. Three-dimensional transperineal/translabial ultrasound was performed with GE Voluson systems (Model S6 or newer; General Electric, Boston, MA, USA) with the patient in the supine position and an empty bladder during rest, maximum Valsalva, and pelvic floor muscle contraction. The plane of minimal hiatal dimensions at maximal pelvic floor muscle contraction was used for tomographic imaging of the puborectalis component of the LAM, with an interslice interval of 2.5 mm. As previously described [18], a full avulsion, unilateral or bilateral, was defined as an interruption between bone and muscle observed in at least three central tomographic slices (Fig. 2). Trauma, whether unilateral or bilateral, was quantified using a tomographic trauma score (TTS) that ranks LAM injury on a 0‒12 scale, with “0” = no injury and “12” = complete bilateral avulsion [19].

Three board-certified urogynecologists, each with 15‒20 years of experience interpreting images who were blinded to randomization and delivery details, independently reviewed the ultrasound images using GE 4dView software (General Electric). Differences in diagnosis were resolved through review of the images in conference to achieve agreement by at least two readers. If ultrasound images were indeterminate for LAM status the participant was asked to return for a repeat ultrasound.

### Outcome Measures

The primary effectiveness endpoint was the rate of pelvic muscle injury, defined as complete LAM avulsion diagnosed by transperineal ultrasound of the pelvic floor anatomy at 3 months postpartum. Secondary effectiveness measures included rates of partial LAM avulsion, perineal lacerations, obstetric anal sphincter injuries (OASIS) [20], conversion to C-section owing to arrest of the second stage of labor, and duration of the second stage of labor. The primary safety outcome was incidence of adverse events (AEs) through 3 months postpartum.

### Statistical Analysis

Categorical variables were compared using the Chi-squared test, with reversion to Fisher’s exact test for evaluating the primary effectiveness outcome when any value on 2 × 2 contingency tables was ≤ 5. Continuous variables were compared using Student’s *t* test when the data were normally distributed, and the Wilcoxon rank sum test when not normally distributed. Two-tailed *p* values < 0.05 were considered indicative of significant differences. Statistical software was SAS v.9.4 (SAS Institute, Cary, NC, USA). The original power calculation was based on a primary endpoint of the incidence of both partial and full avulsions. With feedback from the FDA regarding the uncertainty related to the healing of partial avulsions, the primary efficacy endpoint was chosen to be full LAM avulsion rate at 3 months. This change rendered our original power analysis (based on 80% power to detect a ≥ 50% reduction in full and partial LAM injury with device use, at a two-tailed 0.05 type 1 error rate) invalid. A per-protocol population was analyzed consisting of those participants who delivered vaginally, completed treatment defined as device expansion to at least 6.7 cm, and returned for 3-month follow-up ultrasound. During the time delay between randomization and planned device placement, some device-arm participants underwent a C-section for fetal intolerance to labor or experienced rapid progression to complete cervical dilation, precluding device placement; these participants were excluded from analysis. The safety population included individuals who were randomized and received the device.

## Results

**Fig. 3:**
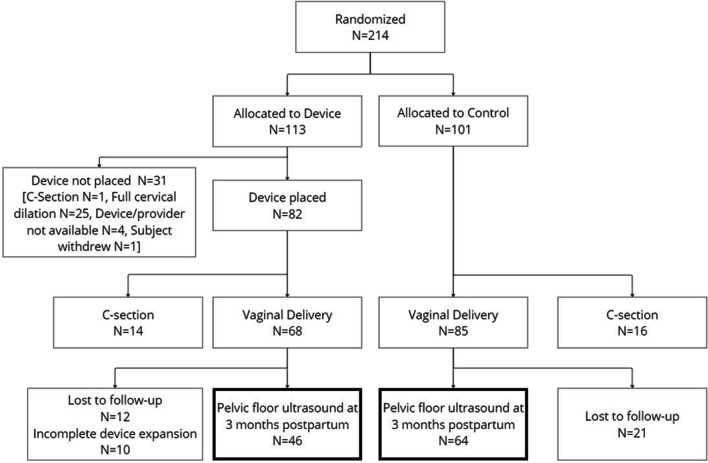
Enrollment and study progression. A total of 214 women were randomized to Device (*n* = 113) or Control (*n* = 101) arms. Of 113 Device assignees, 82 had a device placed, of whom 68 delivered vaginally. Of 101 Control participants, 85 delivered vaginally. At 3 months, 110 participants, 46 Device subjects who received full device treatment and 64 Controls, underwent ultrasound for the per-protocol analysis

Of 492 individuals who were screened and consented to participate, 214 participants were enrolled in the study and randomized to the Device (*n* = 113) and Control (*n* = 101) arms. The study flow diagram is shown in Fig. 3. Demographics were equivalent between study arms (Table 1). Of 113 participants randomized to the treatment arm, 82 (72.6%) had an IPD placed; 31 were precluded from device placement because of full cervical dilation (*n* = 25), no available device and/or trained provider (*n* = 4), subject decision (*n* = 1), or C-section (*n* = 1). There were 14 C-section conversions in the Device arm after IPD treatment. Of the 68 treated participants who delivered vaginally and completed device treatment, 46 (67.6%) returned for 3-month postpartum ultrasound. Of the 101 Control participants, 85 experienced a vaginal delivery and 64 of these (75.3%) returned for the 3-month ultrasound. Timing from delivery until ultrasound imaging was a median 106 days (interquartile range 93‒140 days) in the Device arm and 103 days (interquartile range 92‒129 days) in the Control arm.

**Table 1:** Participant demographics

Parameter	Device, *N* = 82	Control, *N* = 101	Nominal *p* value
Age, years, mean ± SD (range)	29.5 ± 6.0 (18, 43)	29.7 ± 5.4 (18, 44)	0.813
Ethnicity, self-reported, *n*/*N*			
Not Hispanic or Latina	84.1 (69/82)	79.2 (80/101)	0.448
Hispanic or Latina	15.9 (13/82)	16.8 (17/101)	0.999
Unknown	0.0 (0/82)	2.0 (2/101)	0.503
Not reported	0.0 (0/82)	2.0 (2/101)	0.503
Race, self-reported, %, *n*/*N*			
American Indian or Alaska Native	0.0 (0/82)	1.0 (1/101)	0.999
Asian	14.6 (12/82)	14.9 (15/101)	0.999
Black or African–American	13.4 (11/82)	12.9 (13/101)	0.999
Native Hawaiian/Other Pacific Islander	0.0 (0/82)	0.0 (0/101)	0.999
White	61.0 (50/82)	58.4 (59/101)	0.763
Other/not reported	11.0 (9/82)	12.9 (13/101)	0.820
Body Mass Index, kg/m^2^, mean ± SD (range)	32.9 ± 6.2 (21.6, 49.9)	32.5 ± 7.8 (23.1, 77.7)	0.706

Labor and delivery characteristics are shown in Table 2. Average gestational age in both groups was 39.2 weeks. Labor was induced in 87.8% and 76.2% of participants in the Device and Control groups respectively (*p* = 0.045). There was no difference between the two groups in rates of episiotomy, perineal tears, or instrument-assisted vaginal delivery. The rate of C-section conversion for active phase arrest of or fetal intolerance to labor, rate of C-section for second-stage arrest, median duration of the second stage of labor for vaginal deliveries, and birthweights were all similar between groups. Median IPD treatment time was 62 min.

**Table 2:** Labor and delivery characteristics

Parameter	Device, *N* = 82	Control, *N* = 101	Nominal *p* value
Gestational age, weeks, mean ± SD (range)	39.2 ± 1.1 (36.0, 41.1)	39.2 ± 1.1 (36.4, 41.6)	1.00
Induced labor, % (*n*/*N*)	87.8 (72/82)	76.2 (77/101)	0.045
Epidural use, % (*n*/*N*)	100.0 (82/82)	100.0 (101/101)	1.00
Second-stage labor duration, min, mean ± SD (range), median (IQR)	112 ± 118 (10, 861), 75 (38‒148)	120 ± 98 (13, 753), 93 (56‒156)	Non-normal* 0.17
Birth weight, g, mean ± SD (range)	3,428 ± 336 (2,353, 4,224)	3,335 ± 461 (2,183, 4,508)	0.14
Apgar ≥ 7 at 1 min, % (*n*/*N*)	80.5 (66/82)	83.2 (84/101)	0.64
Apgar ≥ 7 at 5 min, % (*n*/*N*)	96.3 (79/82)	95.1 (96/101)	0.67
C-section delivery, % (*n*/*N*)	17.1 (14/82)	15.8 (16/101)	0.82
Reason for C-section			
Active phase arrest, % (*n*/*N*)	8.5 (7/82)	5.9 (6/101)	0.50
Abnormal or indeterminate fetal heart rate tracing, % (*n*/*N*)	4.9 (4/82)	3.0 (3/101)	0.50
Second stage arrest, % (*n*/*N*)	3.7 (3/82)	6.9 (7/101)	0.33
Vaginal delivery	82.9 (68/82)	84.2 (85/101)	0.82
Vaginal delivery only			
Second-stage labor duration, min, mean ± SD (range), median, (IQR)	93 ± 72 (10, 298), 74, (38–133)	105 ± 68 (13, 308), 80, (52–147)	Non-normal* 0.16
Perineal tears, % (*n*/*N*)	75.0 (51/68)	80.0 (68/85)	0.46
Any instrumentation use, % (*n*/*N*)	11.8 (8/68)	11.8 (10/85)	1.00
Forceps use	2.9 (2/68)	2.4 (2/85)	
Vacuum	8.8 (6/68)	9.4 (8/85)	
Episiotomy	1.5 (1/68)	2.4 (2/85)	0.70
Birth weight, g, mean ± SD (range)	3,404 ± 362 (2,353, 4,224)	3,288 ± 464 (2,183, 4,508)	0.095
Apgar ≥ 7 at 5 min, % (*n*/*N*)	97.1 (66/68)	95.3 (81/85)	0.58

*IQR* interquartile range

*Data that were not normally distributed were compared using Wilcoxon rank sum test instead of Student’s *t* test

Of 82 participants in the Device group, 46 (56.1%) received complete treatment with the device (expansion to ≥ 6.7 cm), delivered vaginally, and returned for 3-month postpartum pelvic floor ultrasounds. Among the 101 participants in the Control group 64 (63.4%) delivered vaginally and returned for the postpartum ultrasound (Fig. 2). The rate of complete avulsions was significantly reduced in the Device group (0.0%, 0/46) versus Controls (10.9%, 7/64; *p* = 0.040; Table 3). No differences existed between groups in the rate of partial avulsions or combined (partial + complete) avulsions. Anal sphincter injuries occurred at similar rates and severities in both groups.

**Table 3:** Three-month ultrasound results

Parameter, % (*n*/*N*)	Device, *n* = 46	Control, *n* = 64	Nominal *p* value
Complete avulsion	0.0 (0/46)	10.9 (7/64)	0.040
Partial avulsion	15.2 (7/46)	10.9 (7/64)	0.569
Complete or partial avulsion	15.2 (7/46)	21.9 (14/64)	0.465
OASIS (per ultrasound^a^)	10.9 (5/46)	11.5 (7/61^b^)	0.999
OASIS grade			
3a	4.3 (2/46)	4.9 (3/61)	
3b	6.5 (3/46)	6.6 (4/61)	
3c or 4	0.0 (0/46)	0.0 (0/61)	

*OASIS* obstetric anal sphincter injuries

^a^Clinically diagnosed OASIS rates were 10.9% (5/46) and 6.3% (4/64) in Device and Control subjects respectively (*p* = 0.487)

^b^Ultrasound images from three Control participants provided inadequate visualization of potential sphincter defects and were excluded from analysis

Rates of total maternal labor and delivery- and/or device-related, nonserious AEs were similar in the two arms (Table 4). There were 5 cases (6.1%) of vaginal abrasion or bruising recorded in the Device arm versus none in the Control arm (*p* = 0.012). Chorioamnionitis was diagnosed in 9 (11.0%) participants in the Device arm and 6 (5.9%) participants in the Control arm (*p* = 0.33).

**Table 4:** Adverse events (*AEs*)

AEs, % (*n*/*N*)	Device, *N* = 82	Control, *N* = 101	Nominal *p* value
Maternal non-serious AEs			
Any AE	79.3 (65/82)	79.2 (80/101)	0.99
Abnormal bleeding^a^	21.9 (18/82)	12.9 (13/101)	0.10
Pain/discomfort	22.0 (18/82)	14.9 (15/101)	0.22
Urinary urgency/leakage	9.8 (8/82)	10.9 (11/101)	0.80
Chorioamnionitis	11.0 (9/82)	5.9 (6/101)	0.22
Vaginal abrasion/bruising	6.1 (5/82)	0.0 (0/101)	0.012
Maternal serious AEs			
Any serious AE	19.5 (16/82)	8.9 (9/101)	0.038
Postpartum hemorrhage (> 1,000 ml)	6.1 (5/872)	6.9 (7/101)	0.82
Preeclampsia, with severe features	3.7 (3/82)	0.0 (0/101)	0.05
Other infection^b^	3.7 (3/82)	0.0 (0/101)	0.05
Retained placenta	2.4 (2/82)	0.0 (0/101)	0.11
Other serious AE^c^	2.4 (2/82)	1.0 (1/101)	0.11
Other bleeding	1.2 (1/82)	0.0 (0/101)	0.27
Abnormal intrapartum bleeding	1.2 (1/82)	0.0 (0/101)	0.27
Endometritis	1.2 (1/82)	0.0 (0/101)	0.27
Other pain	0.0 (0/82)	1.0 (1/101)	0.37
Neonate serious AEs			
Any serious AE	2.4 (2/82)	4.0 (4/101)	0.57
Fetal distress	1.2 (1/82)	0.0 (0/101)	0.27
Respiratory distress	1.2 (1/82)	2.0 (2/101)	0.69
Brachial plexus injury	0.0 (0/82)	2.0 (2/101)	0.20
Hypoxic ischemic encephalopathy	0.0 (0/82)	2.0 (2/101)	0.20

^a^Abnormal bleeding includes, intrapartum, postpartum 500‒1,000 ml, rectal, and abnormal menstrual bleeding

^b^Other infections included a single event each of cholecystitis, appendicitis, and mastitis in the Device group during the 3-month follow-up

^c^Other serious AEs included a single-event each of appendectomy and transfusion-related acute lung injury in the Device group and a single occurrence of ovarian cyst in the Control group

Sixteen participants (19.5%) in the Device arm and 9 participants (8.9%) in the Control arm reported at least one serious AE (SAE; *p* = 0.038); however, nearly all SAEs in the Device arm were judged to be unrelated to using the IPD device during vaginal delivery (Table 4). This included 3 participants who developed preeclampsia with severe features and 3 individuals with non-obstetrical-related infections (cholecystitis, appendicitis, mastitis). The proportion of Device participants (12 out of 82) and Control participants (7 out of 101) with SAEs that were related to either the device and/or labor and delivery were similar (*p* = 0.09). The single device-related SAE was a vaginal laceration seen after device removal, associated with significant bleeding, and the need for extensive suturing. Postpartum hemorrhage was noted in 5 Device participants (6.1%) and 7 Control participants (6.9%) (*p* = 0.82).

The rate of neonatal SAEs was similar in the two arms (Table 4). There was 1 reported occurrence of respiratory distress and 1 of fetal distress in the Device arm, and 2 cases each of respiratory distress, brachial plexus injury, and hypoxic ischemic encephalopathy in the Control arm. There was no difference between groups with regard to the proportion of neonates born with an Apgar score ≤ 7 at 5 min.

All 110 participants who returned for 3-month follow-up ultrasounds also completed the Pelvic Floor Impact Questionnaire-7 (PFIQ-7), Pelvic Floor Distress Inventory-20 (PFDI-20), and satisfaction survey (Table 5) [17]. The mean PFIQ-7 impact score was 40% lower (better) in Device participants (12.4 points) than in Controls (20.6 points); however, this difference did not reach statistical significance (*p* = 0.24). Similarly, although the mean PFDI-20 distress score was 17% lower (better) in the Device group (30.9 points) versus Controls (37.2 points), these differences were similar (*p* = 0.35). Participants in the device arm provided an assessment of their experience and whether they would recommend the device to others on a scale of 0‒10, where higher scores indicate more favorable response. The mean response was 7.7, demonstrating that participants were generally satisfied with the use of the device during their labor.

**Table 5:** Three-month patient-reported outcomes

Parameter, mean ± SD (range)	Device, *N* = 54	Control, *N* = 66	Nominal *p* value
Pelvic Floor Impact Questionnaire-7 score (maximum score = 300)	12.4 ± 38.2 (0.0, 242.9)	20.6 ± 37.7 (0.0, 219.0)	0.24
Bladder or urine	2.9 ± 12.2 (0.0, 81.0)	6.7 ± 15.6 (0.0, 81.0)	
Bowel or rectum	3.1 ± 11.6 (0.0, 81.0)	6.1 ± 14.9 (0.0, 71.4)	
Vagina or pelvis	6.4 ± 17.4 (0.0, 85.7)	7.8 ± 13.1 (0.0, 71.4)	
Pelvic Floor Distress Inventory-20 score (maximum score = 300)	30.9 ± 33.0 (0.0, 149.0)	37.2 ± 39.8 (0.0, 201.0)	0.35
Pelvic Organ Prolapse Distress Inventory 6	9.0 ± 12.8 (0.0, 54.2)	9.3 ± 12.2 (0.0, 45.8)	
Colorectal-Anal Distress Inventory 8	10.4 ± 11.6 (0.0, 40.6)	12.4 ± 16.0 (0.0, 71.9)	
Urinary Distress Inventory 6	11.5 ± 15.6 (0.0, 66.7)	15.6 ± 17.3 (0.0, 83.3)	

## Discussion

In this pilot study, use of the electromechanical IPD device significantly reduced the risk of full LAM avulsion—no participant in the Device-treatment group experienced full LAM avulsion. This result supports the concept that the elasticity of the pelvic floor muscles can be gradually enhanced through mechanical stretching prior to delivery of the fetal vertex, resulting in reduced likelihood of LAM avulsion from its attachment at the pubic rami. This is consistent with the findings of a previous feasibility study using an earlier version of the device [12].

The mechanism for reducing pelvic floor injury with use of the IPD device is based on the properties of the pelvic floor muscles [20, 21]. Research in sports medicine indicates that muscle and connective tissues are viscoelastic and elasticity is strain-rate dependent, i.e., the slower a muscle is stretched, the less likely it is that there will be injury [22]. In the second stage of labor, the pelvic floor muscles experience rapid distension. MRI-based computer modeling shows that the distal-most pelvic muscles comprising the levator complex must lengthen by a factor of 3‒4 during fetal head crowning [10, 11]. Classic teaching in obstetrical care emphasizes the role of the clinician in limiting the rate of pelvic floor stretch during delivery by supporting the perineum and controlling the speed of head expulsion [23]. Paradoxically, the extreme dilation of the pelvic tissues during childbirth creates resistance that impedes and slows delivery of the baby, and simultaneously over-stretches and injures pelvic tissues. The currently investigated electromechanical IPD device slowly prepares the pelvic floor muscles by maximizing their stretch prior to delivering the fetal head.

A major benefit of reducing levator ani injury risk during vaginal delivery is the probable reduced risk of developing POP later in life [3, 6, 7], an association that appears to strengthen over decades [24]. Indeed, 55% of women with LAM avulsion develop POP within 6‒17 years after first delivery compared with 21% of women without LAM avulsion [3]. Injury to the LAM may also increase the likelihood of developing stress incontinence, although that relationship is equivocal [25]. To exclude potential pre-existing pelvic floor injury in our cohort, we only enrolled nulliparous individuals. Second vaginal deliveries do not seem to have a deleterious effect on LAM biometry and structural integrity [2, 26]. Future longitudinal studies will determine if the IPD device can be safely and effectively used in second pregnancies, or whether first pregnancy IPD treatment provides adequate protection against LAM avulsion injuries in subsequent vaginal deliveries.

The IPD device had a favorable safety profile when used as an adjunct during the first vaginal delivery. Nonserious vaginal abrasion/bruising identified in five Device participants (6.1%) was considered a device-related minor AE; there were no cases of vaginal abrasion or bruising reported in the Control arm. The study protocol instructed investigators to examine the vaginal walls for any evidence of tissue injury after device removal. This may have introduced bias because no intrapartum examination of the vagina was required for the Control arm.

A significant difference was recorded between the two groups for SAE occurrence. However, of the 16 participants in the Device group with a maternal SAE, 3 were preeclampsia with severe features, 2 were cases of retained placenta, and 3 were related to non-obstetrical infection. None of these complications was device related. There was no significant difference in the incidence of L&D- and device-related SAEs between the Device and Control groups (14.6% vs 6.9% respectively, *p* = 0.09).

The only device-related SAE reported was a vaginal laceration seen after device removal that required extensive suturing to achieve hemostasis. Of note, that participant had a nonreassuring fetal heart tracing that required maternal lateral repositioning multiple times. In addition, her vaginal mucosa was very friable and denuded, which contributed to the challenges in controlling bleeding. Subsequently, the instructions for use of the device were changed to limit the number of maternal repositioning maneuvers with the device in place and to exclude individuals identified with friable vaginal tissue prior to randomization.

The rates of chorioamnionitis and endometritis were statistically similar in the Device and Control arms, suggesting that the presence of an intravaginal device in labor does not enhance the risk of ascending bacterial microbes and infection. The number of cervical examinations performed during labor is an independent risk factor for the development of clinical chorioamnionitis [27]. Placement of the IPD does not require a digital examination of the vagina or cervix. Once the device is placed, there is no requirement for additional internal examinations. Unlike intrauterine pressure catheters that are inserted through the cervical canal and into the uterine cavity providing a potential pathway for ascending bacteria [28], the IPD is placed only 4‒5 cm within the vaginal canal.

We theorized that pre-stretching of the pelvic floor with the device would mimic the perineal massage technique that has been shown to potentially reduce the risk of serious perineal lacerations [29]. The incidence of ultrasound-diagnosed OASIS in the Device and Control arms was similar. Taken together with the significant reduction in complete LAM avulsion rate, the IPD device appears effective for stretching and preparing the pelvic floor muscles to accommodate rapid delivery of the newborn, but does not have a discernable impact on perineal/anal sphincter injury rates. Participants who employed the IPD device reported favorable impressions and experiences, with no negative impact on quality of life.

A primary study limitation was the mid-study protocol change to a more stringent primary efficacy outcome (i.e., full avulsion rate instead of combined partial + full avulsion rate). Although this change invalidated initial power calculations of sample size requirements, this pilot study was nonetheless able to discern a statistically significant benefit of treatment on reducing the incidence of full avulsions. The reasoning behind this change was that low-grade partial avulsions may potentially heal postoperatively and thus have limited clinical significance, whereas a fully avulsed LAM is unlikely to spontaneously reattach after injury. A pivotal clinical trial (Clinicaltrials.gov identifier NCT03973281) is currently enrolling subjects to confirm the findings of this pilot study. Another limitation was the necessary partial-blinding design, which may have introduced unintentional bias in recording peripartum events, including AE rates. This did not impact the blinded assessment of LAM avulsion on ultrasound images. Several Device participants converted to C-section or experienced rapid progression to full cervical dilation before randomization; thus, LAM injury findings of this study are not generalizable to the subgroup of individuals with a rapid first-stage labor.

Study recruitment was aimed at including populations with a history of receiving inequitable care for pelvic floor disorders [13, 30]. Participants approximated the proportion of Black individuals and exceeded that of Asian people currently present in the diversifying USA populace [13], and our findings of reduced LAM avulsion with IPD use are likely to apply to a diverse group of labor patients. A study currently ongoing with larger planned enrollment should permit detailed subgroup analyses.

In conclusion, the electromechanical IPD device reduced the prevalence of full LAM avulsion, a surrogate endpoint for developing POP. Counseling pregnant individuals on the risk of long-term sequelae of vaginal delivery is not typically provided during antenatal care. Other than avoiding use of obstetrical forceps, there are few tools available for preventing LAM avulsion. Incorporating the IPD into obstetrical practice may prove beneficial for protecting against pelvic floor injuries responsible for future POP.

## Declarations

### Data Sharing Statement

The anonymized datasets used and/or analyzed during the current study are available from the corresponding author upon reasonable request.

### Prior Presentation

These data have not been previously presented or published.
